# Effect of Training Eye Care Clinical Guideline for ICU Patients on Clinical Competence of Eye Care in Nurses

**DOI:** 10.1155/2021/6669538

**Published:** 2021-01-12

**Authors:** Zakieh Momeni Mehrjardi, Samaneh Mirzaei, Mohsen Gohari, Abbass Hafezieh, Khadijeh Nasiriani

**Affiliations:** ^1^Master of Intensive Care Nursing, International Campus of Shahid Sadoughi University of Medical Sciences, Yazd, Iran; ^2^Student Research Committee, Shahid Rahnemoun Hospital, Shahid Sadoughi University of Medical Sciences, Yazd, Iran; ^3^Ophthalmology Department, Geriatric Ophthalmology Research Center, Shahid Sadoughi University of Medical Sciences, Yazd, Iran; ^4^Nursing Department, Nursing and Midwifery Research Center, Maternal and Newborn Health Research Center, Shahid Sadoughi University of Medical Sciences, Yazd, Iran

## Abstract

**Introduction:**

Sight is one of the most important and vital human senses. Lack of proper eye care (EC) in anesthetized patients can lead to serious ocular complications and even vision loss. Insufficient knowledge, attitude, and skills of nurses are considered as a barrier to providing EC in the intensive care unit (ICU). The aim of the present study was to determine the effect of training EC clinical practice guidelines for ICU patients on nurses' knowledge, attitude, and practice of EC.

**Methods:**

This was an interventional study with a pre-post design performed on 60 ICU nurses. For the experimental group, EC clinical guideline training was performed for anesthetized patients in three sessions. The data collection tool included nurses' clinical competence of the EC questionnaire with a possible score range of 0–86. This tool consists of three domains, including knowledge (0–18), attitude (0–28), and practice (0–40), which was completed in a self-assessment manner before and three months after the training program. Data analysis was carried out using SPSS16. *Findings*. The mean scores of knowledge, attitude, and practice after the intervention in the experimental and control groups were 15.03 ± 2.72 and 11.11 ± 3.50, 25.65 ± 3.47 and 22.07 ± 3.08, and 33.88 ± 4.14 and 28.5 ± 55.08, respectively, which were statistically significant (*P* ≤ 0.001). Also, the total score of clinical competence of EC after the intervention in the experimental and control groups was 74.56 ± 7.93 and 61.74 ± 9.66, which showed a significant difference (*P* ≤ 0.001).

**Conclusion:**

Training nurses based on EC clinical guidelines for anesthetized patients can improve the knowledge, attitude, and practice of ICU nurses. Evidence-based EC practice requires continuous training based on clinical guidelines and EC practice monitoring by nursing managers according to EC clinical guideline for an anesthetized patient.

## 1. Introduction

Sight is one of the most important and vital human senses through which human beings receive most of their information and cognition [1]. Intensive care unit (ICU) patients undergo treatment for life-threatening conditions [2] and are rarely hospitalized due to ocular injury [3], but the risk of ocular complications increases in ICU-admitted clients with reduced levels of consciousness due to loss of the natural protective mechanisms of the eyes, such as reduced tear production and blinking reflexes [4].

Although ICU in-patient care requires support for all body systems, most nursing care focuses on life-threatening problems; this can reduce the health care team's focus on other organs, including eyes [5, 6] so that even a simple procedure such as eye care (EC) by nurses is easily neglected [7]. Most ICU patients, on the other hand, need nursing care to maintain the natural and pathophysiological health of their eyes and should not experience complications associated with a lack of standard care [3]. The absence of appropriate care can lead to serious ocular complications and even vision loss. However, maintaining the health status and integrity of the eye surface is vital to prevent corneal injury and infection [5, 8]. If EC is performed regularly, the incidence of ocular surface complications will be reduced to 8%, which indicates the importance of EC in ICU [5]. EC in ICU is not a priority and becomes a side issue because it focused on the treatment of organ failures [3, 9]. So, lack of amount of EC research is a common matter across the globe [3, 10]. Also nursing care uses for EC are not evidence-based in some countries [4, 11].

An essential aspect of nursing care for critically ill patients is to provide proper EC in ICU [10]. ICU nurses play an essential role in preventing and monitoring eye problems and management. Nurses need to take special care of the patient's eye at the beginning of admission in ICU [9]. In this regard, it is necessary to assess the knowledge and skills of nurses working in ICU in EC care [12]. Some researchers reported that ICU staff do not have sufficient knowledge to provide EC and that lack of knowledge, attitudes, and skills can be considered as a barrier to providing EC [13]. However, sufficient knowledge and skills of ICU nurses play a key role in providing high-quality care [14]. It seems that the education of clinical practice guidelines will lead to evidence-based care. On the other hand, there is a lack of research in improving nurses' knowledge and practice in EC in ICU, especially for patients with loss of consciousness and who are intubated. The present study investigated the effect of eye care clinical guidelines (ECCG) education for ICU patients on the nurse's knowledge, attitude, and practice in EC. The results can be used to improve and provide EC in ICU.

## 2. Methods

This was an interventional study with a pre-post design that measured and compared scores before and after the ECCG training of ICU nurses divided into control and experimental groups. This study was performed in the Intensive Care Units of Shahid Dr. Rahnemoon Teaching Hospital (the main hospital for neurosurgery), Yazd, Iran. Inclusion criteria were nurses with at least a bachelor's degree and working in ICUs for at least 6 months. Exclusion criteria included part-time employment in ICUs, transfer of nurses to other departments, absence of nurses in more than one of the class sessions, and incomplete questionnaire. The sample size was calculated as 30 people per group according to the following formula: 1-*β* = 80%, 1-*α* = 95%, *P*1 = 1.04%, *P*2 = 26% and a 15% attrition rate was considered. Therefore, a total of 60 nurses were selected using a purposive sampling method and were divided into experimental and control groups using random allocation software.

The data collection tool included demographic characteristics form including age, sex, and work experience in the ICU, type of ICU, and level of education, passing the infection control course, and passing the specialized critical care course. The data collection tool was the eye care clinical competency (ECCC) questionnaire designed by Ebadi et al. (2015). This tool consists of three domains: knowledge, attitude, and practice. The knowledge domain consists of 18 five-point items, each with one correct answer. The possible score range is 0–18. The attitude domain consists of 7 items that are scored using a 5-point Likert scale ranging from Very High, High, Moderate, Low, and Very Low. The possible score range is 0–28. The practice domain consists of 10 items scored based on a five-point Likert scale ranging from Always, Often, Sometimes, Rarely, and Not at all. The possible score range is also 0–40, with higher scores indicating a more favorable situation. The possible score range of total clinical competence was 0–86. Content validity and reliability have been confirmed by internal consistency assessment techniques. Cronbach's alpha used for practice and attitude domains as well as Kuder–Richardson Formula 20 (KR-20) used for the knowledge domain showed that the internal consistency of the questionnaire was satisfactory. Cronbach's alpha was 0.83 for the whole questionnaire [13].

Prior to the classes, knowledge and attitude domains of the EC clinical competency questionnaire were completed by the nurses of the control and experimental groups in a self-assessment manner and the practice section was completed as an observation by an educational supervisor. Necessary shift scheduling was made with the educational supervisor and ward officials in order to enable nurses to participate in the classes. Prior to classes, necessary arrangements such as place, time, educational package, and catering were made. The educational content was presented in the form of lectures, discussions, and questions and answers during three weeks (Table 1). It should be noted that the program content was compiled by reviewing the guideline and related programs [9, 15, 16]. The educational supervisor monitored the nurses in the intervention group during the ward round for compliance with the EC standard after the training sessions. Three months after the end of the training sessions, the questionnaire was reevaluated in two groups. In the experimental group, practice items were examined by the educational supervisor and head nurse in three different work shifts for each nurse. Also, practice items were examined in one shift in the control group. To be studied, the contents of the training package were provided to the control group after sampling.

The present study was approved by the Research Ethics Committee of Shahid Sadoughi University of Medical Sciences in Yard with the ethics ID : IR.SSU.MEDICINE.REC.1397.039. Written informed consent was also obtained from the participating nurses after explaining the objectives of the study and the confidentiality of information.

Data analysis was performed using SPSS 16. Descriptive statistics used included absolute and relative frequency and mean (*M*) and standard deviation (SD). The inferential statistics used included independent *t*-test, paired *t*-test, and chi-square with a 95% confidence interval.

## 3. Findings

A total of 60 ICU nurses (30 in the experimental group and 30 in the control group) were included in the study. It should be noted that one person in the experimental group was excluded from the study (due to not attending training session) and three in the control group (two persons were omitted due to being transferred to other departments and one person due to submitting the incomplete questionnaire). Data analysis was performed on 27 people in the control group and 29 people in the experimental group.

Based on the findings of the study, the mean age and work experience of the participants in the experimental and control groups were 36.41 ± 5.42 and 8.21 ± 5.79 years and 34.6 ± 93.26 and 8.07 ± 5.51 years, respectively. Other demographic information of the participants is presented in Table 2. Chi-square and independent *t*-test showed that the two groups were homogenous in terms of age, sex, work experience in the ICU, type of ICU, level of education, passing the infection control course, and passing the specialized critical care course (Table 2).

The findings revealed no significant difference between the experimental and control groups in terms of the mean scores of knowledge, attitude, practice, and total score of ECCC before the intervention; however, the independent *t*-test reported such significant difference after the intervention (*P* ≤ 0.001). There was also a significant difference between the experimental and control groups in terms of the mean score of total ECCC after the intervention (*P* ≤ 0.001). A paired *t*-test also revealed a significant change in the mean scores of knowledge, attitude, and practice before and after the intervention in the experimental group (*P* ≤ 0.001). There was no significant change in the mean scores of knowledge and attitude in the control group before and after the intervention (*P*=0.195), (*P*=0.071), but there was a statistically significant difference in practice scores before and after the intervention in the above group (*P* ≤ 0.001). A paired *t*-test also revealed a significant difference between the two groups in terms of the mean score of total ECCC (*P* ≤ 0.001) (Table 3).

## 4. Discussion

The present study investigated the effect of ECCG training on the EC clinical competence in ICU nurses assigned into experimental (clinical EC training) and control groups (routine training). Findings revealed that the majority of the participants in both groups were female, with a bachelor's degree and employed in the neurological ICU. Also, the majority of participants in both groups had passed the infection control course but did not have EC instructions in the ward and had not passed the specialized critical care course. There was also no statistically significant difference between the control and experimental groups in terms of demographic information. Liem (2019) writes that 92.2% (*n* = 13) of ICU nurses reported that they had no previous EC training and all participants acknowledged that they did not have an EC protocol to follow [17].

The mean score of EC clinical competence in all three domains of knowledge, attitude, and practice and the total score of clinical competence was slightly higher than average, and almost the same before the intervention in the experimental and control groups, and there was no statistically significant difference between the two groups in this regard. Considering that the most skilled nurses are employed in ICUs on the one hand, and the eye is considered as an important organ as the sense of sight, on the other hand, ICU nurses are expected to have a high level of EC knowledge, attitude, and practice for anesthetized patients. In a study of EC knowledge and practice of ICU nurses, Alghamdi et al. (2018) reported that the total EC knowledge score of nurses was less than 50% and nurses had satisfactory knowledge about EC of patients undergoing mechanical ventilation; however, nurses showed high attitude towards EC procedures in patients undergoing mechanical ventilation, but this finding necessarily did not mean that they had good EC clinical practice [8]. Vyas et al. (2018) investigated EC knowledge and practice patterns of ICU nurses to determine their level of knowledge and practice pattern about exposure keratopathy in patients with mechanical ventilation. Although there was a high level of knowledge about EC in general, nurses had no appropriate practice pattern, and training programs are needed to improve nurses' EC knowledge and practice [18]. In a study on the EC knowledge and practice of nurses, Khalil et al. (2019) also showed that although nurses have sufficient EC knowledge, they have poor EC practice [19]. Based on the findings of the present study and other studies, there seems to be a need for training EC to ICU nurses. Of course, it can be due to poor compliance or inadequate supervision.

The findings also demonstrated an increase in the mean score of ECCC in all three domains and the total score of clinical competence in both groups in the postintervention phase. However, there was a greater increase and a significant difference in the experimental group; therefore, it seems that the ECCG training has increased the knowledge, attitude, and practice of nurses. In this regard, in a study of development and validation of EC training program, Cho et al. (2017) reported a significant increase in EC-related knowledge, awareness, and practice score after the implementation of the training program [16]. In a study of the effect of EC training on the incidence of corneal surface disorders in ICU patients, Demirel et al. (2014) wrote that exposure keratopathy significantly decreased before and after training courses and that raising EC awareness among nurses helps improve EC in ICU patients with a reduced level of consciousness undergoing ventilation [20]. Also, in a study of the effect of training EC protocol to 260 ICU nurses, Fashafsheh et al. (2013) found a significant reduction in the incidence of exposure keratopathy in patients after training and also showed a significant difference in the score of knowledge and practice of nurses after training [3]. In this regard, Dawson (2005), while emphasizing the provision of information on surface eye diseases and clinical symptoms of EC in ICU, states that increasing such awareness would increase nurses' interest in paying attention to eye complications and EC [21]. In Liem's (2019) study, 75% of the participants referred to a lack of training as a barrier to EC in ICU [17]. These studies confirm the findings of the present study regarding the positive effect of training on nurses' knowledge and attitude and subsequent practice improvement. These studies are consistent with the results of the present study and confirm the effectiveness of training.

The results also showed that the mean score of knowledge, attitude, practice, and total score of clinical competence increased significantly in the experimental group in the postintervention phase as compared to the preintervention phase. This finding shows that training has affected the knowledge, attitude, and practice of patients.

The results, nevertheless, showed no significant increase in the postintervention mean score of knowledge, attitude, practice and the total score of clinical competence in the control group; however, such increase was significant in the practice domain and the total clinical competence score, which suggests that the nurses in the experimental and control groups were working in similar departments; this has led to covert training of control group nurses and thus an improvement in their practice score.

One of the limitations of the present study is that the nurses of the experimental and control groups worked directly in ICU which can be effective in learning EC and potential change in practice in the control group. Another limitation of the study was the small sample size. There were also problems with scheduling nurses' shifts to attend classes. Therefore, attempts were made to schedule programs on less busy working days, such as the last working day of the week, so that it would be more possible to participate in the classes. In this regard, the educational supervisor coordinated with the head nurses about the program. It is thus recommended to carry out future studies on the effect of implementing the ECCG on the incidence of eye disorders in ICU.

## 5. Conclusion

Overall, the EC training program has been able to improve the EC clinical competency of ICU nurses providing care to anesthetized patients; however, it seems that in order to achieve the highest score, it is necessary to implement a continuous training program. Therefore, it is recommended to consider ECCG training as one of the topics of continuing training for nurses. In this regard, in order to maintain the effectiveness of training programs, they should be repeated based on the latest changes in the ECCG. Moreover, the ECCG should be taught to undergraduate and postgraduate nursing students. Nursing managers should also pay attention to the content of the ECCG to improve the quality of monitoring the quality of EC nursing services to pave the way for the implementation of the contents of the ECCG. It is also recommended to improve the quality of nursing care and evidence-based practice in other areas based on approved clinical guidelines.

## Figures and Tables

**Table 1:** Scheduling of EC training session for the experimental group.

≠	Lecturer	Educational content
First session	Optometrist	Educational objectives, description of eye structure and function, eye disorders and pathophysiology of eye diseases, risk factors for eye diseases and eye examinations, eye evaluation, eyelid position, and drug medication.

Second session	Professor of nursing	The incidence of eye diseases, the importance of EC, eye problems in ICU patients, and a review of the latest research on eye disorders in the intensive care unit.

Third session	Educational supervisor	EC methods, eye hygiene, methods for closure of the eyelids, and eye assessment methods and eyelid position and EC in practice.

**Table 2:** Demographic characteristics of participants.

Group variable		Experimental group		Control group		*P* value^*∗*^
		Frequency	%	Frequency	%	
Sex	Male	11	37.93	8	29.62	0.35
	Female	18	62.07	19	60.38	

Level of education	BA	25	86.2	26	96.29	0.62
	MA	4	13.8	1	3.71	

Ward	General ICU	13	44.82	11	40.74	0.23
	Neurosurgery ICU	16	55.18	16	59.26	

Passing the specialized critical care course	Yes	2	6.89	2	7.41	0.94
	No	27		25	92.59	

Passing the infection control course	Yes	22	75.87	17	62.96	0.29
	No	7	24.13	10	37.04	

*∗* : chi-square test.

**Table 3:** Comparison of mean scores of knowledge, attitude, practice, and total score of ECCC in the participants before and after the intervention.

Group	Knowledge			Attitude			Practice			Competency		
	*M*±SD (0–18)		*P* value^*∗*^	*M*±SD (0–28)		*P* value^*∗*^	*M*±SD (0–40)		*P* value^*∗*^	*M*±SD (0–86)		*P* value^*∗*^
	Before	After		Before	After		Before	After		Before	After	
Control	10.59 ± 3.58	11.11 ± 3.5	0.195	21.88 ± 3.52	22.07 ± 3.08	0.711	25.85 ± 4.1	28.55 ± 5.08	0.0001	58.33 ± 8.44	61.74 ± 9.66	0.001
Experimental	10.17 ± 2.4	15.03 ± 2.27	0.001	23.10 ± 3.55	25.65 ± 3.47	0.001	25.75 ± 3.77	33.88 ± 4.14	0.001	59.03 ± 6.48	61.74 ± 9.66	0.001
*P* value^*∗∗*^	0.6	0.001		0.2	0.001		0.93	0.001		0.73	0.001	

^*∗*^Paired *t*-test. ^*∗∗*^Independent *t*-test.

## Data Availability

The data to support the findings of this study are available from the corresponding author upon request.

## Abbreviations

EC: Eye care
ECCG: Eye care clinical guideline
ECCC: Eye care clinical competency
ICU: Intensive care unit
M: Mean
SD: Standard deviation.
